# Macrorheology of cystic fibrosis, chronic obstructive pulmonary disease & normal sputum

**DOI:** 10.1186/1465-9921-10-63

**Published:** 2009-07-06

**Authors:** David J Serisier, Mary P Carroll, Janis K Shute, Simon A Young

**Affiliations:** 1 Department of Respiratory Medicine, Mater Adult Hospital, Brisbane, Australia; 2 Dept of Respiratory Medicine, Southampton General Hospitals NHS Trust, UK; 3 Institute of Biomedical and Biomolecular Sciences, School of Pharmacy and Biomedical Sciences, University of Portsmouth, UK; 4 School of Pharmacy, The University of Auckland, Auckland, New Zealand

## Abstract

**Background:**

Prior microrheologic assessments of selected, microlitre plugs of cystic fibrosis (CF) sputum suggest no intrinsic rheologic abnormality. However, such analyses may not be representative of CF sputum as a whole. We therefore reassessed this question using whole sputum macrorheology. Additionally, we wished to further explore the relationships between sputum rheology, inflammation and infection.

**Methods:**

Dynamic oscillatory macrorheometry was performed on whole expectorated sputum from stable adults with CF (n = 18) and COPD (n = 12) and induced sputum from normal controls (n = 7). Concomitant sputum inflammatory mediator levels were measured in CF and COPD samples. Sputum collected from CF subjects (n = 6) at commencement and completion of intravenous antibiotic therapy for an infective exacerbation was also assessed.

**Results:**

CF sputum neutrophil elastase activity (NE) was significantly related to degree of sputum purulence (p = 0.049) and correlated significantly with measures of sputum viscoelasticity (r = 0.696, p = 0.008 for storage modulus G' at 9 Hz). There were significant differences in viscoelasticity between subject groups when samples were compared irrespective of appearance/degree of sputum purulence. However, the macrorheology of mucoid CF sputum did not differ from normal sputum (eg median (range) G' at 9 Hz 2.25 (0.79, 3.26) vs 2.04 (1.4,4.6) Pa, p = 1). In contrast, mucoid COPD samples demonstrated significantly greater viscoelasticity (G' at 9 Hz 4.5 (2.4, 23) Pa) than sputum from both CF (p = 0.048) & normal subjects (p = 0.009). Antibiotic therapy during exacerbations was associated with significant reductions in CF sputum viscoelasticity, with mean (SD) G' at 9 Hz decreasing from 28.5 (11.5) Pa at commencement to 6.4 (4.6) Pa on day 7 (p = 0.01).

**Conclusion:**

The macrorheologic properties of whole, mucoid CF sputum are not different from normal, confirming the results of prior microrheologic studies. Instead, CF sputum viscoelasticity is related to secondary infection, decreases with intravenous antibiotic therapy and correlates with inflammation. In contrast, COPD sputum demonstrates inherently greater viscoelasticity, providing a novel target for potential therapeutic interventions.

## Background

The exact pathophysiology linking cystic fibrosis transmembrane conductance regulator (CFTR) dysfunction with CF pulmonary disease remains unclear, although the popular airway surface liquid (ASL) volume theory [1,2] predicts that dehydrated, thickened respiratory secretions are integral to mucociliary stasis and the subsequent development of CF airway pathology. Indeed, thickened, tenacious respiratory secretions are a ubiquitous feature of CF that are widely considered to represent dehydrated respiratory secretions with abnormal viscoelasticity [3-5].

However, prior investigations have failed to demonstrate abnormal viscoelasticity of CF sputum when compared with canine tracheal mucus [6]. While some prior studies have not shown CF sputum to have greater viscoelasticity than asthma, bronchiectasis and chronic bronchitis sputum [7,8], others have shown significantly higher viscoelasticity of CF than COPD sputum [9]. There are a number of potential limitations of some previous investigations, including the use of high shear rates that may disrupt the mucus gel network of respiratory mucus [7,8], the comparison of CF sputum with non-human (canine) 'normal' mucus [6] and the analysis of microlitre quantities of selected sputum plugs that raise the possibility of sample bias given the heterogeneity of CF sputum [6].

Simple visual inspection of CF sputum reveals its obvious, marked heterogeneity. This visual variability is confirmed in sputum measures including inflammatory markers [10] and rheology, with work from our and other groups showing large variations in viscoelasticity when analysing separate aliquots from the same sputum samples (coefficients of variation of up to 150% for aliquots from the same samples) [11,12]. This extreme variability must create concern over the validity of analyses that use small selected sputum portions.

In the context of these validity concerns, the disparity between current concepts around the pathogenesis of CF lung disease and its expression in the form of sputum rheology led us to re-examine the rheology of CF sputum using whole sputum samples to reduce the risks of sample bias and improve reproducibility. We hypothesised that macrorheologic analysis of whole CF sputum, when compared to normal human sputum (obtained by induction), would enable the postulated intrinsic rheologic abnormalities of CF sputum to be demonstrated. Additionally, we further investigated the contribution of infection to the rheology of CF sputum from individual subjects by evaluating the effects of intravenous antibiotic therapy upon sputum rheology. Finally, relationships between sputum inflammatory markers and sputum rheology were investigated.

## Methods

### Subjects and Study Design

This was a prospective study comprising firstly a cross-sectional component in which COPD and CF subjects produced a single spontaneously expectorated sputum sample, while normal subjects produced a single sputum specimen following sputum induction (SI) with 4.5% hypertonic saline. A prospective, open interventional component involved analysing sputum samples from CF subjects at the commencement of a course of intravenous antibiotic therapy for an infective exacerbation, and then during and at the completion of the course. The local regional ethics committee provided approval.

All subjects were recruited through the Respiratory Medicine Department of Southampton General Hospital, were current non-smokers over 16 years of age, and provided informed consent. No subject was prescribed mucolytic agents or systemic corticosteroids, and 'stable' subjects had not received supplemental antibiotic therapy in the prior two weeks. Normal subjects (n = 7) were healthy volunteers without respiratory disease, mean (SD) age 31 (7.6) years. Twelve chronic bronchitic COPD subjects, 72 (11) years old and FEV_1 _57.2 (33.9)% predicted, had a history of chronic daily sputum production and were clinically stable with no recent exacerbation of lung disease. These subjects had a significant cigarette smoking history (at least 20 pack years) and irreversible airflow obstruction (FEV: FVC<0.7). Sputum was obtained from 18 clinically stable, adult CF subjects (diagnosed by standard criteria) with chronic pulmonary *Pseudomonas aeruginosa *infection, age 24.9 (7.4) years and FEV_1 _46.6 (13.9)% predicted. For the investigation of the effects of intravenous antibiotic therapy, six CF subjects receiving intravenous antibiotic therapy for an infective exacerbation were included (meeting predefined criteria incorporating ≥ 10% fall in FEV_1 _from baseline in combination with at least two of the following: 1. Increased sputum purulence; 2. Increased sputum volume; 3. Fevers or systemic symptoms; 4. Weight loss of at least 1 kg; 5. New crackles on chest auscultation; 6. New infiltrates on chest radiograph.).

Specimens were collected in sterile, hermetically sealed containers and frozen at -80°C (previous work and our own unpublished data has shown no significant effect of freezing upon the rheological properties of sputum analysed after a single freeze-thaw cycle [13]) and were characterised according to macroscopic appearance, as mucoid (no evidence of pus), purulent (uniformly purulent whether green or yellow) or mucopurulent (mixed mucoid & purulent components) [6,8].

SI was performed according to the standardised protocol of the European Respiratory Society taskforce [14] using an ultrasonic nebuliser (Devilbiss, Somerset, USA).

### Rheologic Analysis

Sputum was thawed at room temperature for one hour and salivary contamination carefully removed. The whole sample was then gently homogenised with 10 strokes in a 5 mL syringe. High shear forces can disrupt mucus gel structure and influence measured rheology and prior investigators have reported that homogenization may destroy mucus structure [15]. However, those reports employed vigorous homogenisation including "blending by vigorous stirring for six minutes" [15] in contrast to the gentle homogenisation technique used in the current studies. Our group has recently shown that this homogenisation procedure has no effect upon sputum pore size [16] and that there was no significant difference between the rheological characteristics of the gently homogenised sputum specimens and selected nonhomogenised aliquots from the same sputum samples [11]. Rheology was performed using a Carri-Med CSL 100 controlled-stress rheometer (Carri-Med Ltd, Dorking, England) operating in dynamic oscillatory mode employing a 4 cm stainless steel parallel-plate geometry. Following homogenisation, the sample was loaded in the rheometer geometry and any entrapped air bubbles removed. A solvent trap containing distilled water was used to prevent dessication and analyses were performed at 20°C to reduce enzymatic sample degradation. Samples equilibrated for 30 minutes prior to all experiments to enable relaxation to their original gel structure. Torque sweeps were performed initially (0.1 – 10 Pa) to determine a stress at which the response of the elastic modulus (G') and loss modulus (G'') was within the linear viscoelastic region for the sample. Logarithmic frequency sweeps (0.1–10 Hz) were then performed in triplicate at the determined shear stress and displacement.

Data obtained from these experiments were used to calculate rheologic parameters within the linear viscoelastic region, with particular interest in storage modulus G', and dynamic viscosity η', at the frequencies 0.3 Hz and 9 Hz. This provided both high and low frequency data, with 9 Hz approximating to ciliary beat frequency.

### Determination of Sputum Inflammatory Markers

Thirteen CF and seven COPD sputum samples had concomitant inflammatory mediator levels determined. After thawing the samples on ice, salivary contamination was removed by pipette and the sample diluted (1:1 mL per g) with phosphate buffered saline (PBS, Gibco, UK) for solubilisation of mediators. The samples were thoroughly mixed (vortex mixer for five minutes then orbital plate mixer for 20 minutes, on ice) then centrifuged at 20,000 g, at 4°C for 20 minutes. The supernatant was aspirated and used to determine neutrophil elastase (NE) activity, IL-8, myeloperoxidase (MPO) and terminal complement complex (TCC) levels. Commercially available enzyme-linked immunosorbent assay (ELISA) kits were used to measure total IL-8 (Pelikine compact, Mast Diagnostics, Liverpool, UK) in supernatant samples diluted at either 1:1000 or 1:10000 with assay dilution buffer, and TCC (Quidel, California, USA) in supernatant samples diluted 1:2 with assay diluent. MPO was measured using a commercially available radioimmunoassay kit (Pharmacia, Uppsala, Sweden) in supernatant samples diluted at 1:1000 with PBS. NE activity was measured in samples diluted 1:10 with assay buffer as previously described [17].

### Data Analysis

The one-sample Kolmogorov-Smirnov Test indicated that rheological parameters assessed in normal and COPD samples were normally distributed. However, for CF samples, a number of parameters were not normally distributed and non-parametric tests were therefore used for comparisons. The Kruskal-Wallis test for multiple independent samples was used to evaluate differences between the groups, and Mann Whitney U test to compare groups individually. Correlations between variables used Spearman's test for non-normally distributed variables. For serial studies of the same subjects, parametric analyses were used. P values less than 0.05 were considered significant. Where large numbers of outcome measures were assessed, a *Bonferroni *adjustment has been employed such that significance at the 5% level was indicated for p values < 0.05/m where m is the number of comparisons made. SPSS for Windows release 11.0.0 (SPSS Inc, Chicago) was used.

## Results

### CF Sputum Purulence and Sputum Macrorheology

**Figure 1:**
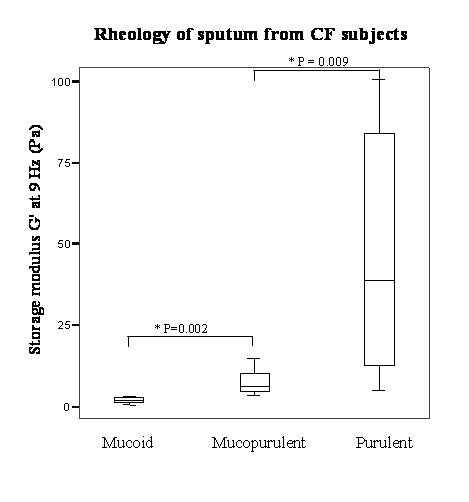
**Box and whisker plot comparing mucoid, mucopurulent and purulent sputum from CF subjects for storage modulus**. (Illustrating the graded increase in elasticity measures at 9 Hz occurring with increasing macroscopic purulence of sputum from stable CF subjects. Mucoid n = 4, mucopurulent n = 10, purulent n = 8. * by Mann-Whitney U-test).

As expected, CF sputum inflammatory mediator levels were positively related to the degree of purulence assessed macroscopically (e.g. median NE levels increased significantly from 2.75 mu/μl for mucoid to 13.85 for mucopurulent and 31.95 for purulent, p = 0.049 by Kruskal-Wallis; see Table 1), consistent with the prior findings of Stockley *et al *in subjects with chronic bronchitis and bronchiectasis [18]. Significant differences in sputum rheology were also seen between CF sputum samples, for all measures of viscoelasticity (G' and η' at both 0.3 and 9 Hz, Log G* at 1 rad s^-1 ^and Tan δ), according to the degree of sputum purulence (see Figure 1 for indication).

**Table 1:** Inflammatory mediator levels in sputum from CF and COPD subjects.

	**Cystic fibrosis**			
	**NE **(mu/μl)	**IL-8 **(X10^2 ^ng/mL)	**TCC **(ng/mL)	**MPO **(mg/L)

**Mucoid **(n = 1)	2.75	0.85	0	34.30

**Mucopurulent **(n = 6)	13.85 (8.31–42.89)	1.22 (0.89–3.94)	0 (0–0.73)	53.47 (39.13–213.35)

**Purulent **(n = 6)	31.95 (14.94–62.13)	2.11 (0.91–34.35)	30.36 (0–326.44)	139.41 (51.44–238.15)

P value *	***0.049***	0.141	0.089	0.169

	**COPD**			

**Mucoid **(n = 4)	0 (0–1.33)	6.52 (3.51–8.14)		

**Mucopurulent **(n = 2)	4.5 (0–9)	9.77 (5.93–13.6)		

**Purulent **(n = 1)	35.11	6.15		

P value *	0.20	0.85		

(Values presented are median (range). NE – neutrophil elastase activity, IL-8 – interleukin-8, TCC – terminal complement complex, MPO – myeloperoxidase. * by Kruskal-Wallis Test).

### Relationship Between CF Sputum Inflammatory Mediators and Macrorheology

There were significant positive correlations with sputum viscoelasticity measures for NE activity (r = 0.696, p = 0.008 for G' at 9 Hz; r = 0.735, p = 0.004 for η' at 9 Hz), MPO (r = 0.741, p = 0.006 for G' at 9 Hz; r = 0.72, p = 0.008 for η' at 9 Hz) and TCC (r = 0.631, p = 0.028 for G' at 9 Hz; r = 0.653, p = 0.021 for η' at 9 Hz) but no significant relationships for IL-8. These relationships extend prior data showing correlations between CF sputum infection and cough transportability [19] and between CF sputum inflammation and sputum transportability [20].

### Comparison Between Patient Groups

**Figure 2:**
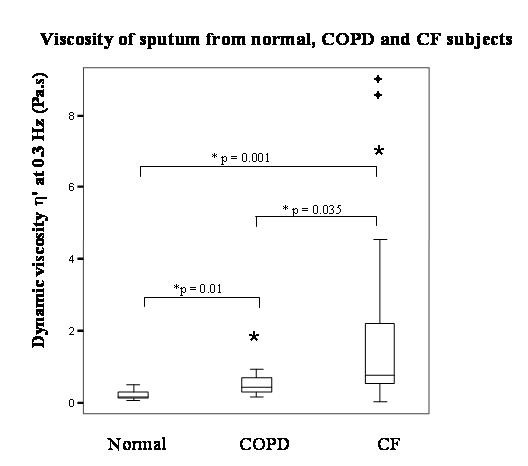
**Box and whisker plot comparing rheology of sputum (irrespective of macroscopic appearance) from normal, COPD and CF subjects, for dynamic viscosity**. (As indicated, significant differences were observed between all groups for dynamic viscosity η' at 0.3 Hz. Normal n = 7, COPD n = 12, CF n = 18. * by Mann-Whitney U Test).

There were large, statistically significant differences seen between normal, COPD and CF sputum when sputum was compared between groups irrespective of sputum appearance/degree of sputum purulence (p ≤ 0.01 by Kruskal-Wallis Test for all analysed rheological parameters, G' and η' at both 0.3 and 9 Hz, Log G* @ 1 rad s^-1 ^and Tan δ; Figure 2 provides indication of the magnitude of the differences, for dynamic viscosity).

**Figure 3:**
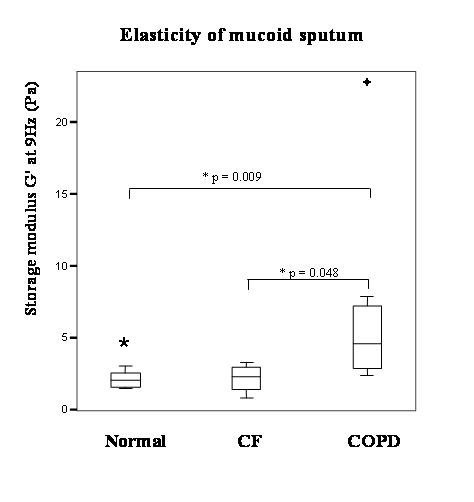
**Box and whisker plot comparing rheology of mucoid sputum from normal, COPD and CF subjects for storage modulus**. (No significant differences were observed between normal and CF sputum for storage modulus G' at 9 Hz. Normal n = 7, CF n = 4, COPD n = 8. * by Mann-Whitney U Test).

When analyses were restricted to macroscopically similar, mucoid ('uninfected') sputum, there were no differences in rheology between CF (mean percent predicted FEV_1 _52.5%) and normal sputum. Mucoid COPD sputum showed higher values than both CF and normal sputum for all parameters of viscosity and elasticity (see Table 2 and Figure 3 for an indication of the magnitude of differences).

**Table 2:** Macrorheologic characteristics of mucoid sputum from CF, COPD and normal subjects.

	**CF (n = 4)**	**Normal (n = 7)**	**COPD (n = 8) **	P value * (CF v COPD)	P value * (N v COPD)
**G' @ 0.3 Hz (Pa)**	0.54 (0.03–1.1)	0.39 (0.02–1.6)	1.4 (0.22–13.8)	0.109	0.054

**G' @ 9 Hz (Pa)**	2.25 (0.79–3.26)	2.04 (1.4–4.6)	4.5 (2.4–23)	***0.048***	***0.009***

**η' @ 0.3 Hz (Pa)**	0.24 (0.04–0.38)	0.15 (0.04–0.49)	0.41 (0.16–1.8)	0.154	***0.04***

**η' @ 9 Hz (Pa)**	0.03 (0.02–0.04)	0.025 (0.023–0.034)	0.04 (0.025–0.1)	0.109	***0.002***

Log G* (1)	0.89 (-0.08–1.2)	0.76 (-0.06–1.3)	1.3 (0.7–2.2)	0.073	***0.023***

**Tan δ (1)**	0.87 (0.65–2.6)	0.93 (0.43–2.5)	0.55 (0.2–1.6)	0.214	0.094

(No significant differences between CF and normal sputum, p > 0.68 for all measures. Values presented are median (range). (1) -- @ 1 Rad s^-1^, G' – storage modulus, η' – dynamic viscosity, tan δ – loss tangent, Log G* – mucus rigidity index. Log G* & Tan δ are dimensionless. * by Mann-Whitney U-test).

For the COPD samples levels of inflammatory mediators were mostly undetectable in these mucoid samples (median [range] neutrophil elastase levels for mucoid samples 0 [0–1.3] mu/μl), once again confirming the low levels of inflammation present in COPD sputum of mucoid macroscopic appearance [18]. Not surprisingly therefore, no relationships were seen between sputum inflammatory mediators and viscoelasticity for COPD samples.

### Effect of intravenous antibiotic therapy upon sputum rheology

**Figure 4:**
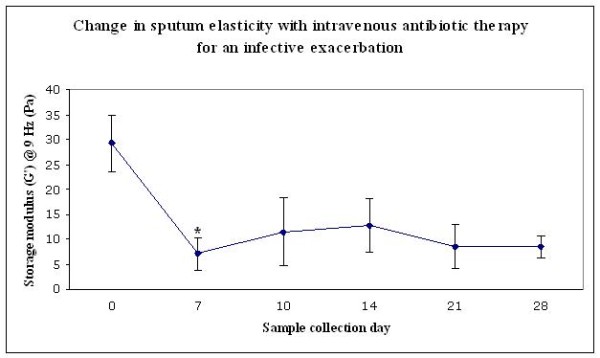
**Change in CF sputum rheology with therapy for an infective exacerbation (Change in mean storage modulus G' at 9 Hz is depicted**. Error bars indicate SEM. * p = 0.01 for the comparison with day 0 by paired T-test; Using a Bonferroni adjustment, significance at the 5% level is indicated by a p value of 0.01).

Two subjects were only able to provide sputum at commencement of the study and were therefore excluded from subsequent analysis. Mean FEV_1 _for the remaining subjects was 43%. There were significant decreases from day 0 to day 7 in several rheologic parameters including storage modulus G' at both 0.3 Hz (p = 0.006) and 9 Hz (p = 0.01; see Figure 4). It may be appropriate to incorporate a Bonferroni adjustment into these analyses, in which case significance at the 5% level is indicated by a p value of 0.01 (0.5/5).

## Discussion

The current observations extend prior data that have shown relationships between CF sputum bacteriology and cough transportability [19] and between CF sputum inflammatory mediators and mucus transportability [20]. To our knowledge, the current data are the first to demonstrate direct positive relationships between CF sputum inflammatory mediator levels and sputum viscoelasticity. Furthermore, consistent with the importance of purulence/infection in determining the magnitude of sputum viscoelasticity in CF subjects, applying an effective anti-infective therapy (intravenous antibiotics) was associated with large reductions in sputum elasticity. The original primary hypothesis of this study was that our alternative, large volume, whole sputum technique would allow us to prove differences between CF and normal sputum rheology by attenuating the variability associated with selected sputum plugs. However, our data using macrorheologic analysis support the findings of prior microrheologic analyses [6] that rheologic abnormalities of CF sputum are acquired in relation to infection/inflammation rather than being intrinsic.

Other recent lines of evidence accord with our data suggesting that rheologic abnormalities of CF sputum are secondary abnormalities due to established infection and inflammation. For example, if respiratory mucus were inherently abnormal in CF, nasal mucociliary clearance would be impaired in CF children before the onset of significant sinonasal disease, however recent data refute this [21]. Additionally, Bush *et al *recently showed no differences in sputum rheology between subjects with CF and those with primary ciliary dyskinesia, suggesting the importance of common secondary (infection, etc) rather than primary events in the development of abnormal sputum properties [22].

The current data therefore do not support the notion that rheologically abnormal, dehydrated CF sputum (developing as a result of ASL volume loss) is the initial determinant of the mucociliary stasis that is subsequently associated with bacterial infection and progressive airways obstruction in CF. Our data are more consistent with alternative primary models of CF pathophysiology, such as those implicating excessive inflammation [23] or abnormal susceptibility to respiratory infection [24,25], including the recently described oxidative antimicrobial system that appears inactive in the CF airway [26].

The current analysis, using dynamic oscillatory rheometry, did confirm significant intrinsic rheologic abnormalities of non-infected sputum from chronic bronchitic COPD subjects. The increased viscoelasticity of uninfected COPD sputum is likely to be related to increases in mucin concentration within COPD airway secretions [27] associated with goblet cell hypertrophy and submucosal gland hyperplasia [28]. The recent demonstration of increased quantities of mucins within COPD sputum (and significant reductions in the amount of MUC5B and MUC5AC in CF sputum) provides further evidence to support this [29]. It is likely, therefore, that future effective mucolytic agents in COPD will need to either solubilise (or dilute) mucins in the airways or reduce mucin production from airway epithelia. Macrolide antibiotics may represent a potentially effective therapeutic option, with *in vitro *and animal data demonstrating the ability of these agents to attenuate mucin synthesis and mucus secretion [30,31] – the effect of macrolides upon the macrorheology of sputum from chronic bronchitic subjects requires further evaluation.

A number of potential limitations of this study need consideration. Firstly, our comparison of non-purulent respiratory secretions relies upon a small number of CF samples. Few CF subjects produce mucoid secretions, and this created substantial difficulties in collecting such samples. Furthermore, it could be argued that those who produce mucoid secretions are likely to have milder disease (and milder genotypes), however these subjects had a mean percent-predicted FEV_1 _of only 52.5%. Furthermore, we found no relationships between FEV_1 _and any sputum rheological parameter when analysing the full CF data set (consistent with prior work) [14] and there was no difference in sputum rheology between those CF subjects with FEV_1 _< 50% predicted and those with FEV_1 _≥ 50% predicted (data not shown). Secondly, the sputum induction (SI) process may have influenced the rheology of normal sputum *in vivo*, given that the *in vitro *incubation of sputum with hypertonic saline for 30 minutes has been shown to reduce the mucus rigidity index [32]. However, if SI had resulted in any significant reduction in sputum viscoelasticity, this would be likely to have increased the difference between CF and normal sputum, thereby strengthening our finding of no difference. One recent study showed no significant effect of SI upon the rheology of respiratory mucus from non-expectorating normal subjects although did provide data suggesting that SI alters other physical properties of sputum [33]; however, findings from this study are predicated on the assumption that analysis of 'normal' respiratory secretions collected directly from endotracheal tubes at surgery is the 'gold standard' for normal human respiratory mucus.

Others have attempted to overcome some of the problems that are inherent in collecting and evaluating clinical specimens from subjects with and without respiratory disease by evaluating the rheology of secretions obtained from models of the CF airway. Baconnais *et al *found no differences in ion composition or viscosity between airway liquid from normal and CF fetal tracheal xenografts [34].

In summary, using an alternative whole sputum macrorheologic technique, these results confirm that COPD sputum manifests inherent increases in viscoelasticity while there is no inherent rheological abnormality of CF sputum. Rather, CF sputum purulence (related to secondary infection and inflammation) dominates abnormalities of sputum rheology, and intravenous antibiotic therapy improves these parameters. The early initiation in CF subjects of therapies to limit the development of chronic bacterial infection and inflammation, are likely to be critical for attenuating the development of rheologically altered secretions and their later downstream effects on CF airway function. In contrast, stable COPD subjects with chronic sputum production are unlikely to derive rheologic benefit from standard anti-inflammatory or anti-infective therapies and efforts to develop therapies that solubilise mucins or reduce mucin production from airway epithelia require further evaluation. Macrolide antibiotics may therefore offer attractive therapeutic potential in chronic bronchitic COPD subjects.

## Abbreviations

ASL: airway surface liquid; BMI: body mass index; CF: cystic fibrosis; CFTR: cystic fibrosis transmembrane conductance regulator; COPD: chronic obstructive pulmonary disease; ELISA: enzyme-linked immunosorbent assay; FEV_1_: forced expiratory volume in the first second; FEV_1_%: FEV_1 _as a percentage of the predicted value; FVC: forced vital capacity; G': storage modulus; a measure of elasticity; G'': loss modulus; a measure of viscosity; IL-8: interleukin-8; Log G*: mucus rigidity index; Log _10 _(G'' +G'); calculated index of total resistance; MPO: myeloperoxidaseNE: neutrophil elastase; η': dynamic viscosity; a measure of viscosity that accounts for the frequency of the applied stress; PBS: phosphate buffered saline; SI: sputum induction; Tan δ: The loss tangent; the ratio of G''/G'; TCC: terminal complement complex.

## Competing interests

DJS has no conflict of interest; MPC has no conflict of interest; JKS has no conflict of interest; SAY has no conflict of interest. The authors declare that they have no competing interests.

## Authors' contributions

DJS designed the study, recruited subjects, performed laboratory procedures, analysed the data and drafted the manuscript; MPC supervised the project and reviewed the manuscript; JKS supervised the study and laboratory procedures and reviewed the manuscript; SAY supervised the study and laboratory procedures and reviewed the manuscript. All authors read and approved the final manuscript.
